# Digital affordances of AI chatbots in nursing education: a systematic review of learning gains and gaps in the evidence

**DOI:** 10.3389/fmed.2026.1832598

**Published:** 2026-04-24

**Authors:** Shuling Wei, Wei Wei, Guanyang Zou

**Affiliations:** 1 Faculty of Applied Sciences, Macao Polytechnic University, Macao, Macao SAR, China; 2 Center for Research Management and Services, Guangxi Open University, Nanning, China; 3 School of Public Health and Management, Guangzhou University of Chinese Medicine, Guangzhou, China

**Keywords:** affordance, artificial intelligence, chatbot, learning gains, nursing education, systematic review

## Abstract

**Background:**

Digital affordances refer to the possibilities provided by digital environments for learners. In the context of nursing education, artificial intelligence (AI) chatbots currently offer multimodal learning approaches and demonstrate various possibilities for digital actions. Therefore, exploring the digital affordances of AI chatbots in nursing education is crucial for the continuous advancement of the field.

**Objective:**

To evaluate the digital affordances of AI chatbots in nursing education, focusing on the relationship between digital affordances and learning gains.

**Methods:**

We employed affordance theory to conceptualize the potential actions of AI chatbots and utilized a taxonomy of affective, behavioral and cognitive learning gains to conduct a systematic review in nursing education.

**Results and conclusions:**

A total of 25 studies were identified in this systematic review. The geographical distribution of the studies is mainly in Asia. The most used study designs were quantitative designs (*n* = 12) with sample sizes between 16 and 457. The duration of these studies is usually short, ranging from a few hours to 3 months. The included studies reported several digital affordances of AI chatbots in nursing education, including assistance provision, personalization, human-like conversing, distilling information, and fostering familiarity. However, four digital affordances-facilitation, enriching information, context identification, and ensuring privacy-still lack empirical support. The evidence for the digital affordances of AI chatbots in nursing education was dominated by cognitive learning gains (such as learning achievement, critical thinking, and problem solving) and followed by affective (such as learning interest, self-efficacy, and enjoyment) and behavioral learning gains (such as engagement, diagnostic skills and clinical practice). However, several studies reported no statistically significant improvement in certain cognitive learning gains, particularly knowledge acquisition and clinical reasoning competency. Similarly, limited evidence was found for improvements in learners’ confidence and satisfaction. These findings suggest that the current evidence remains inconclusive. Future research should employ longer study durations and larger sample sizes to further examine the educational impact of AI chatbots.

## 1 Introduction

With rapid advances in generative pretraining transformer (GPT) technology, the application of AI chatbots in nursing education has opened up new possibilities for goal-oriented actions (1–4), sparking extensive academic discussions on their innovative applications (5–7). Previous studies have revealed that AI chatbots facilitate well-designed multimodal conversations that can effectively assist in a variety of areas such as patient intervention (8), postoperative support (9), and psychiatric care (10), which has attracted great interest in nursing education research (3). On the other hand, concerns have been raised about the use of chatbots in nursing education. These concerns include: since ChatGPT responses are based on patterns learned from data, issues of accuracy, reliability, and potential bias remain a concern (11); there has been a lack of content and programs for instruction using chatbots (12); and there are concerns about over-reliance on technology, such as issues with plagiarism and limiting critical thinking skills (13). The application of AI chatbots has significantly expanded the scope of nursing education, offering students new goal-oriented action possibilities (i.e., affordances) (14). However, there remains a gap in research regarding the identification of these affordances and their practical applications in nursing education. In other words, how to effectively identify digital affordances in nursing education and in what nursing educational contexts these affordances will emerge remain unclear.

Previous literature has indicated that AI chatbots in nursing education provide nursing students with various digital learning opportunities (15). For example, Han et al. (16) reported that using AI chatbot programs in electronic fetal monitoring significantly improved nursing students’ self-directed learning and nursing skills. Furthermore, Shorey et al. (17) found that simulating patient interaction scenarios with AI chatbots helped nursing students strengthen their communication skills. Another study demonstrated that AI chatbots enhanced students’ learning performance and self-efficacy in obstetric vaccine education by engaging with case-based scenarios (18). In other words, AI chatbots not only support the personalized learning but also promote students’ learning behaviors and skill development, creating new learning opportunities and facilitating the conversion of these opportunities into learning gains.

Despite these potential benefits, the digital affordances of AI chatbots in nursing education still face several challenges. First, students’ cognitive abilities, technology acceptance, and trust in AI chatbots affect the realization of their affordances, as students may not fully understand the opportunities and technical support offered by AI chatbots (12). Second, integrating AI chatbots into traditional nursing education is involved complex and dynamic interactions, and the interplay between teachers, students, and AI chatbots remains insufficiently clarified (19, 20). Lastly, it remains uncertain to what extent the digital affordances of AI chatbot contributes to measurable learning gains, and there is a need to analyze and distinguish these learning gains and their assessment methods (21, 22). Therefore, it is crucial for exploring the new goal-oriented possibilities created by AI chatbots for nursing students and how the digital affordances affect the learning gains.

Due to the increasing popularity of AI chatbots, some empirical studies have explored their effects on students’ learning gains (4). However, there is a lack of in-depth analysis of the similarities and differences in learning gains across different contexts, which raises new questions regarding how to meaningfully and practically assess cognitive, affective, and behavioral learning gains (22–24). For example, Chang et al. (25) demonstrated that AI chatbots not only enhance the cognitive learning performance of nursing students but also improve their behavioral self-efficacy. Additionally, with the rapid advancements in nursing research and the advent of new diseases and medications, nursing professionals are challenged to quickly adapt to evolving medical knowledge, make accurate decisions in real-case scenarios, and master complex nursing skills (1, 17, 26–28). Therefore, incorporating valid and reliable measures of learning gains in nursing education is crucial to ensuring that nursing students effectively master fundamental knowledge, develop critical thinking, enhance problem-solving abilities, and improve communication skills (16, 25, 26). Several studies have utilized AI chatbots in various methods to assess the quality of nursing education (25), such as Han et al. (16), who employed AI chatbots for assessing skills in electronic fetal monitoring, and Shorey et al. (17), who used AI chatbots in patient communication simulations. While these approaches are promising, there remains a need for more robust empirical evidence to validate their effectiveness in accurately measuring the diverse learning gains of nursing students.

Compared with prior reviews on AI chatbots in education, this study offers several distinct contributions. To better understand and interpret the complex interactions between nursing professionals and AI chatbots, this study adopts the perspective of affordance theory (29, 30), conceptualizing the potential actions that AI chatbots enable for educators and students. Concurrently, we have employed a coherent classification approach proposed by Rogaten et al. (24) to review various learning gains and analyze their similarities and differences. In addition, AI literacy is incorporated to account for nursing students’ knowledge, skills, and attitudes required to effectively understand, evaluate, and interact with AI chatbots (31). This approach aims to develop a more comprehensive understanding of the learning gains reported in the literature and to assess the potential of these learning gains as measure of the value of nursing education. The study aims to answer the following research questions:

*RQ1*: What digital affordances do AI chatbots provide in nursing education?

*RQ2*: What types of evidence have been employed to assess the impact of AI chatbots on learning gains in nursing education?

## 2 Method

### 2.1 Search strategies

The most recent search was conducted in the period of November–December 2025 in PubMed and Web of Science (see Table 1). Keywords included combinations of “chatbot,” “nursing,” and “education.” We developed our searching teams based on previous studies, which suggested that nursing education programs may include both health professionals and nurses. Moreover, chatbots in educational programs have been described using various terms, including “Conversational Agents” (32), “AI agent” (33), “artificial intelligent agent” (34) and “ChatGPT” (35). Therefore, we expanded our search in PubMed and Web of Science to include broader terms: “chatbot” OR “Conversational Agent” OR “AI agent” OR “artificial intelligence agent” OR “ChatGPT” (Topic) AND “nurse” OR “nursing” OR “care” OR “health” (Topic) AND “education*” OR “train*” (Topic). We retrieved 178 studies from PubMed and 143 studies from Web of Science.

**Table 1:** Search queries and number of results for each database.

Database	Search terms	Retrieved
PubMed	(“chatbot” OR “Conversational Agent” OR “AI agent” OR “artificial intelligence agent” OR “ChatGPT”) and (“nurse*” OR “nursing*” OR “care” OR “health”) and (“education*” OR “train*”)	179
Web of Science	chatbot or Conversational Agent or AI agent or artificial intelligence agent or ChatGPT (Topic) AND nurse or nursing or care or health (Topic) AND education* or train* (Topic)	144

### 2.2 Inclusion criteria

The inclusion criteria for this study were established using the PIOS framework (see Table 2), which is adapted from the widely used PICOS (participants, interventions, comparators, outcomes, and study design) strategy (36). This decision is justified by the nature of the included studies, many of which do not involve a clearly defined comparator group. Participants include nurses and nursing students. Interventions involve the use of AI chatbots or similar dialogue systems in nursing education, focusing on their impact and effectiveness. The outcomes are concerned with the utilization of AI chatbots as educational or training tools and their effectiveness in nursing education. Study designs encompass all research related to nursing education or training. No direct comparator group was specified.

**Table 2:** Inclusion criteria.

Inclusion criteria	Description
Participants	Nurses, nursing students
Interventions	Involving AI chatbots or similar dialogue systems
Outcome(s)	Utilization of AI chatbots as an educational or training tool
Study designs	Involving nursing education and in service training or professional development

### 2.3 Search process

We conducted a rigorous screening and selection process for this study. Initially, our search identified 323 studies. After removing 88 duplicates, we had 235 unique records. During the title and abstract screening phase, we excluded 205 records for two primary reasons: 188 did not target our demographic of nurses and nursing students, and 17 were review articles. Subsequently, we thoroughly reviewed the full texts of the remaining 30 studies for eligibility. Of these, 5 studies were excluded as they did not focus on nursing education or training. Ultimately, 25 studies met our comprehensive inclusion criteria and were selected for inclusion in the study (see Supplementary file 1).

**Figure 1:**
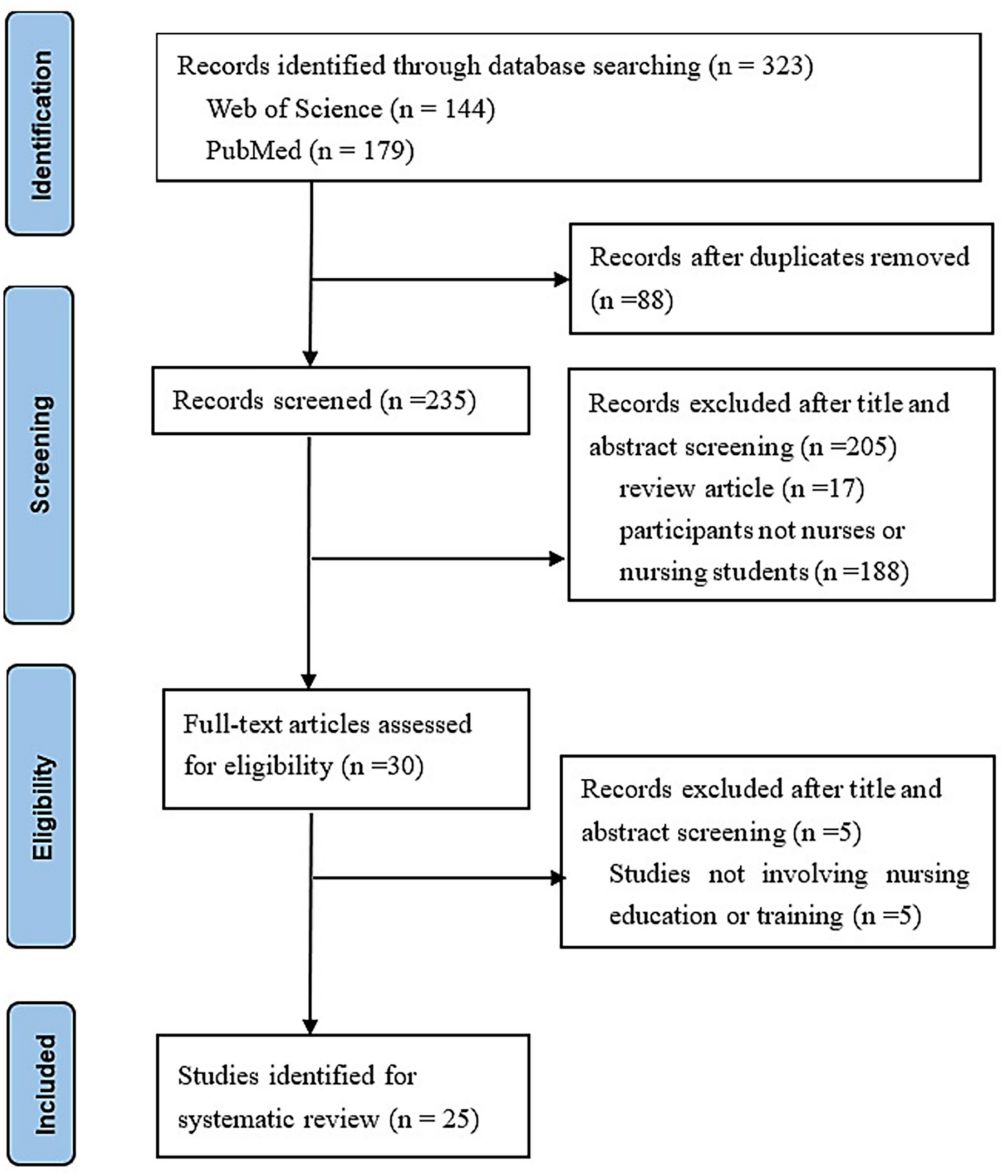
PRISMA flow diagram for systematic review (67).

Detailed methodological characteristics of the included studies, including sample size, intervention duration, and research design, are reported in Supplementary file 1. Further information on the study selection process is provided in the PRISMA flow diagram (Figure 1).

### 2.4 Data analysis and synthesis

The coding system in this study was inspired by Mygland et al. (29) review of state-of-the-art literature on human-AI interactions, which identified and categorized 91 different digital affordances of AI chatbots into nine high-level classes. To improve transparency, a simplified version of the coding framework is presented in Table 3 for reviewers. This table summarizes the main affordance categories used in the analysis. More detailed definitions and coding rules are provided in Supplementary file 2. Furthermore, we applied the ABC learning gains classification approach to analyze digital affordances of AI chatbots across three dimensions: (1) affective learning gains, which can be defined as a change in affect related states during a course (24), illustrated by Han et al. (16) in their evaluation of an AI chatbot’s affective impact on nursing students and nursing trainees’ skills through in a quasi-experiment; (2) behavioral learning gains, which focus more strongly on skills than knowledge (24), demonstrated by Hsu (11) in their study on enhancing medical terminology learning using ChatGPT and Termbot; and (3) Cognitive learning gains, which can be defined as development in knowledge, understanding and cognitive abilities (24), as evidenced by Chang et al. (25) use of a chatbot in a quasi-experiment to improve obstetric vaccination learning, cognitive learning gains.

**Table 3:** High-level digital affordances identified in the literature (29).

High-level affordances	Definition	References
Human-like conversing	Chatbots use AI to simulate conversation, and this changes how nursing students and trainees use software. These tools can understand user intent, make responses, and remember the chat history for later questions	Lunberry and Liebenau (68), Stoeckli et al. (14), Waizenegger et al. (69), Moussawi (70), Lippert et al. (71)
Assistance provision	Chatbots assist in daily tasks and nursing education, offering reminders, functions, info, admin, and personalized support to boost engagement and efficiency	Stoeckli et al. (14), Stoeckli et al. (72), Waizenegger et al. (69), Knote et al. (73), Moussawi (70), Meske and Amojo (74), and Barnett et al. (75)
Facilitation	Chatbots connect nursing students and trainees with organizations, unify system access, and simplify tasks	Stoeckli et al. (14), Knote et al. (73), and Meske and Amojo (74)
Distilling information	Chatbots help summarize information and support mood reflection	Stoeckli et al. (14), Stoeckli et al. (72), Knote et al. (73), and Meske and Amojo (74)
Enriching information	Chatbots use visuals and extra text to share information faster and build stronger connections	Stoeckli et al. (14) and Knote et al. (73)
Context identification	Chatbots understand the context of a conversation, identify needs, give helpful responses, and guide the discussion	Stoeckli et al. (14), Stoeckli et al. (72), Knote et al. (73), Meske and Amojo (74)
Personalization	Chatbots adapt to nursing students and trainees, personalizing responses and tone	Waizenegger et al. (69), Knote et al. (73), Moussawi (70), and Lippert et al. (71)
Fostering familiarity	Chatbots in nursing education build on users’ familiarity with chat apps. Students feel at ease with this interaction, yet unmet expectations may cause dissatisfaction	Moussawi (70)
Ensuring privacy	Chatbots protect privacy and control access. Users share information, so interactions require careful management	Stoeckli et al. (14), Stoeckli et al. (72), Waizenegger et al. (69), and Knote et al. (73)

## 3 Results

This section addressed the aims of the present study in the following order: (1) characteristics of study (including demographic, AI chatbot technology, and research design) (see Table 4); (2) investigated the digital affordances provided by AI chatbots in nursing education; and (3) empirical evidence obtained through the evaluation of AI chatbots.

**Table 4:** Characteristics of study.

*N* = 25	*N*
Sample size	
Less than 20	1
20 to 59	5
60 or more participants	10
No information available	9
Intended user	
Junior nursing students	7
Senior nursing students	14
Nurse practitioners	1
No information available	3
Country/Region	
USA	6
Taiwan	5
Spain	3
Singapore	2
UK	1
Mainland China	1
Türkiye	1
Canada	1
Hungary	1
South Korea	1
Italy	1
Romania	1
Germany	1
AI chatbot technology	
Large language model	10
NLP	9
Voice-controlled AI assistants	2
Knowledge-based system	1
Pattern matching	1
No information available	2
AI chatbot duration	
Long term (monthly)	6
Short term (daily)	5
Medium term (weekly)	4
No information available	10
Research design	
Quantitative (quasi-experiment, cross-sectional comparative study, descriptive statistics, questionnaire)	12
Qualitative (interviews)	2
Mixed methods (document-analysis, usability tests, cross-sectional technology validation study)	4
No information available	7

### 3.1 Demographic

Overall, the sample size of these empirical studies tends to be small, ranging between 16 and 457. The sample sizes in the study were categorized into four groups: less than 20 participants (*n* = 1), 20 to 59 participants (*n* = 5), 60 or more participants (*n* = 10), and a category (*n* = 9) where specific information was not provided. The intended user groups for the AI chatbots included nursing students and nursing professionals. Of the 25 studies, 7 focused on junior nursing students, 14 on senior nursing students, and 1 on nurse practitioners. However, 3 of these studies did not specify their intended user groups. The geographical distribution of the studies was global, with a focus in Asia (5 from Taiwan, 2 from Singapore,1 from Mainland China, 1 from South Korea), North America (6 from the USA, 1 from Canada), and Europe (3 from Spain, 1 from the UK, 1 from Türkiye,1 from Hungary, 1 from Italy, 1 from Romania, 1 from Germany). This international representation highlights the global interest in AI chatbot technology and its applications across different healthcare systems and educational backgrounds. The research encompasses a broad range of user groups, from junior nursing students to senior nursing students and nurse practitioners, demonstrating the potential for AI chatbot applications at various levels of nursing education.

### 3.2 AI chatbot technologies and duration

The results indicate that the usage scenarios of AI chatbots involve dialogue facilitation, intent understanding, and providing instant feedback (37), with both short-term and long-term interventions potentially improving learning gains. Consequently, the AI chatbots in the studies were primarily powered by technologies such as large language model (*n* = 10), NLP (*n* = 9), voice-controlled AI assistants (*n* = 2), knowledge-based systems (*n* = 1), and pattern matching (*n* = 1). Two studies did not clearly specify the key technology used. The duration of these studies varied, ranging from 3 months (17) to a few hours (16). The implementation of AI chatbot applications was categorized into long-term, medium-term, and short-term: 6 studies used the AI chatbot monthly (long-term), 5 studies daily (short-term), and 4 studies weekly (medium-term). Ten studies did not provide specific information regarding the duration of the experiment.

### 3.3 Research design

In reviewing 25 studies on the application of AI chatbots in nursing education, we found that quantitative studies (*n* = 12) primarily addressed the effectiveness of AI chatbots in enhancing nursing skills and knowledge, including medical terminology learning, physical examinations, and electronic fetal monitoring (11, 16, 25). Most of these studies (*n* = 7) employed a quasi-experimental design to assess learning gains using validated scales. In contrast, two qualitative studies measured learning gains through focus group interviews, exploring nursing students’ perceptions, attitudes, and experiences with AI chatbots (17, 26). Mixed-methods studies (*n* = 4) provided an opportunity to evaluate affective, behavioral, and cognitive learning gains, such as in studies examining the impact of AI chatbots on students’ self-regulated learning abilities (28).

### 3.4 The digital affordances of AI chatbots for students and educators in nursing education

This review of 25 studies reveals five high-level digital affordances provided by AI chatbots in nursing education: assistance provision, personalization, human-like conversing, distilling information, and fostering familiarity, as detailed in Table 5. We conceptualize digital affordance as “the possibility of goal-oriented action offered to a specific group of users through a technological object” (38). The concept of digital affordance is therefore relevant and takes into account (1) the competencies and learning goals of nursing students and (2) the characteristics of the AI chatbot (39).

**Table 5:** High-level affordances provided by AI chatbots related to nursing education.

High-level digital affordances	Definition and identified affordances	References
Assistance provision	AI chatbots can perform a variety of assistive tasks, including: (1) speedy assistance, (2) usefulness, (3) executing tasks, (4) live updates, (5) data access, (6) quick answers, (7) error reduction, (8) support seeking	Han et al. (16), Otero-Agra et al. (76), Chang et al. (18), Chang et al. (25), Chang et al. (19), Chow et al. (27), Hsu (11), Chen et al. (26), Sáiz-Manzanares et al. (28), and Kaur et al. (77)
Personalization	AI chatbots can adapt interactions to their users by providing customized responses and adjusting their tone and style, including: (1) personalized learning, (2) interactivity, (3) feedback, (4) adaptivity.	Chang et al. (18), Hsu (11), Hsu et al. (78), Chen et al. (26), Riedel et al. (44), Chang et al. (19), Han et al. (16), and Saiz-Manzanares et al. (28)
Human-like conversing	AI chatbots can generate human-like messages, including: (1) human-like content, (2) conversation mimicry	Chow et al. (27) and Chen et al. (26)
Distilling information	AI chatbots provide users with action possibilities related to distilling information, including: (1) flow maintenance, (2) aggregated data	Han et al. (16), Chang et al. (25), Kaur et al. (77), and Chang et al. (19)
Fostering familiarity	AI chatbots allow user to express their needs directly through a familiar interaction mode, including: (1) emotional connection, (2) comfort growth	Chow et al. (27), Chen et al. (26), and Kaur et al. (77)

In this context, the most common high-level digital affordance provided by AI chatbots is assistance provision, with eight related affordances identified (see Supplementary file 3). Conversely, human-like conversing, distilling information and fostering familiarity were less frequently mentioned. However, the role of AI chatbots seems to focus more on individual support rather than facilitating traditional human-to-human interactions in educational settings. Therefore, the use of AI chatbots in nursing education appears to prioritize practical needs such as assistance provision and personalization over simulating human-like conversing, distilling information and fostering familiarity. It may be worthwhile to consider integrating AI-based chatbots into nursing learning environments to enhance students’ metacognitive awareness and the quality of peer feedback. For instance, researchers have reported that AI-based chatbots can significantly improve learners’ metacognitive awareness (40). In one study, an AI chatbot named Eva was integrated into an online peer review system, significantly improving the quality of student feedback (41). This indicates that AI chatbots may not be fully utilized to offer more interactive and engaging educational experiences.

### 3.5 Relationships between digital affordances and learning gains

**Figure 2:**
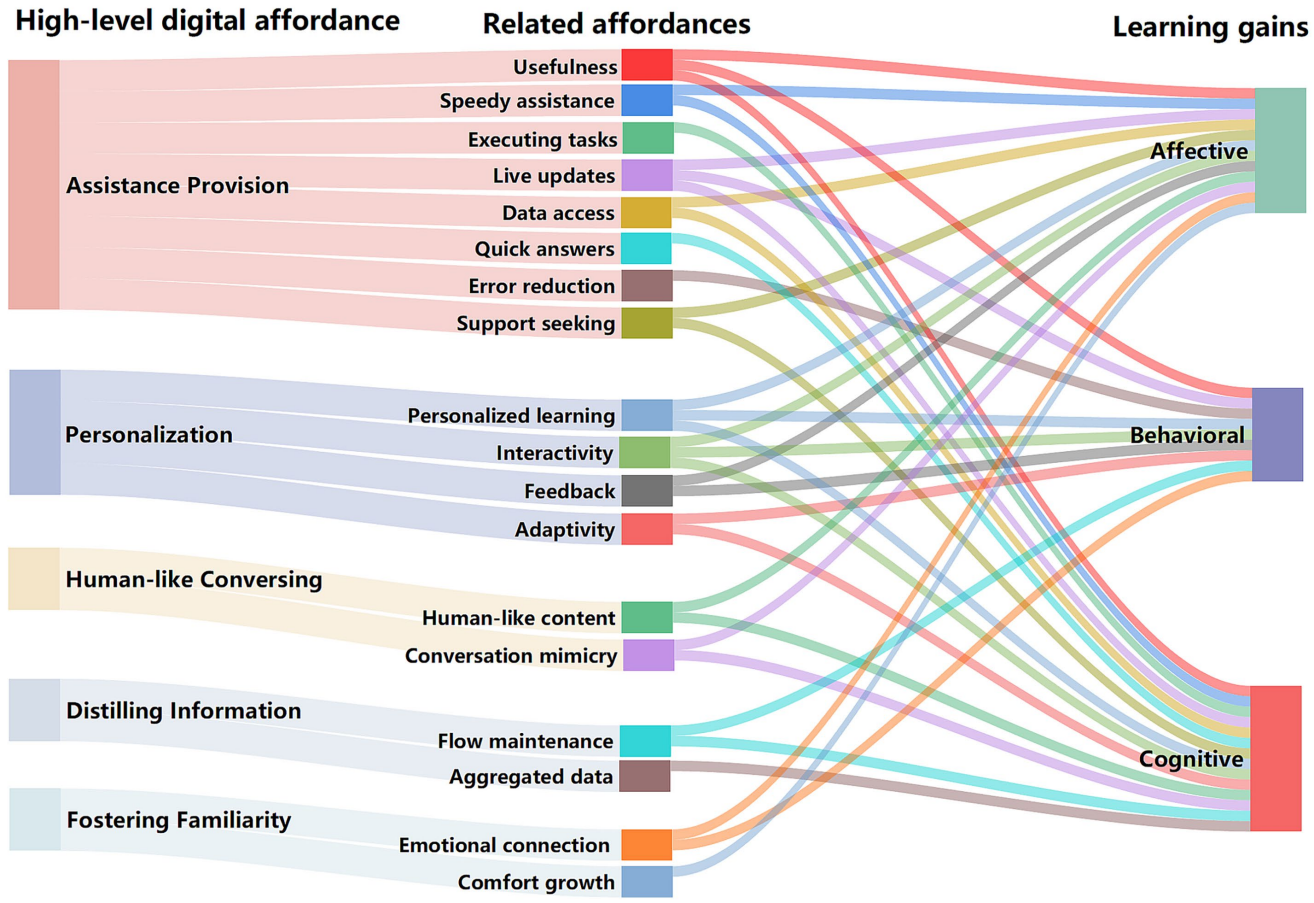
Relationships between digital affordances provided by AI chatbots and learning gains.

An analysis of 18 related affordances revealed an uneven distribution of evidence across learning gains (see Table 6). Specifically, 4 of the 18 related affordances (22.20%) assess a combination of all three learning dimensions (affective, behavioral, and cognitive) (see Figure 2). In contrast, 5 related affordances (27.80%) focus on both affective and cognitive learning gains, 2 related affordances (11.10%) on affective and behavioral, 2 related affordances (11.10%) on behavioral and cognitive, 1 related affordance (5.60%) exclusively on affective learning gains, 1 related affordance (5.60%) on behavioral learning gains, and 3 related affordances (16.60%) on cognitive learning gains. However, there are fewer learning gains have been studied for behavioral change, but some researchers believe that behavioral learning gains deserve further study and that skill development and engagement are as important as knowledge acquisition (37, 42, 43).

**Table 6:** Related affordances identified in nursing education.

High-level digital affordances	Related affordances identified in nursing education	Affective	Behavioral	Cognitive
Assistance provision	Usefulness	✓	✓	✓
	Speedy assistance	✓		✓
	Executing tasks			✓
	Live updates	✓	✓	✓
	Data access	✓		✓
	Quick answers			✓
	Error reduction		✓	
	Support seeking	✓		✓
Personalization	Personalized learning	✓	✓	✓
	Interactivity	✓	✓	✓
	Feedback	✓	✓	
	Adaptivity		✓	✓
Human-like conversing	Human-like content	✓		✓
	Conversation mimicry	✓		✓
Distilling information	Flow maintenance		✓	✓
	Aggregated data			✓
Fostering familiarity	Emotional connection	✓	✓	
	Comfort growth	✓		

#### 3.5.1 Affective learning gains

**Figure 3:**
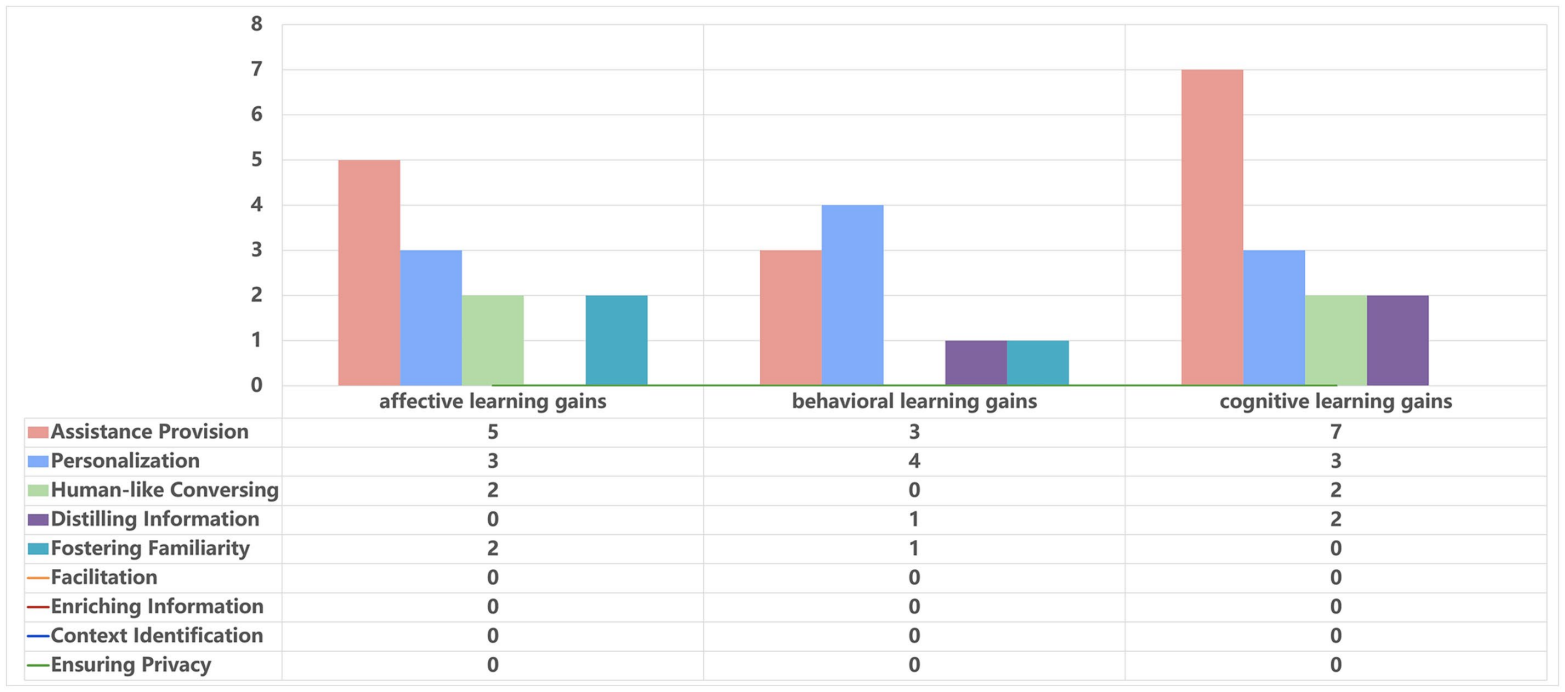
High-level affordances addressing difference dimensions of learning gains.

As presented in Figure 3, we analyzed 18 related affordances identified in nursing education, with 12 addressing affective learning gains including assistance provision (*n* = 5), personalization (*n* = 3), human-like conversing (*n* = 2), and fostering familiarity (*n* = 2). Positive changes in affective learning gains include learning interest (16), self-efficacy (18), satisfaction (25), enjoyment (19), and motivation (11). The assistance provision (*n* = 5) provided by AI chatbots has been shown to enhance students’ learning interest, self-efficacy, satisfaction, and enjoyment (16, 19, 25). For example, Han et al. (16), in their quasi-experimental study, used numerical rating scales to assess the learning interest of 61 nursing students. Compared to traditional methods, the implementation of AI chatbot programs significantly increased nursing students’ learning interest during a 32-min video lecture. Additionally, personalization (*n* = 3) by AI chatbots was found to significantly enhance nursing students’ learning interest and motivation (11, 16). For instance, Chang et al. (25) reported in their pre-post test study that a knowledge-based chatbot, integrated into nursing education, facilitated interactive and personalized learning by adapting to students’ progress and needs. While there are some digital affordances for human-like conversing (*n* = 2) and fostering familiarity (*n* = 2) in nursing education, indicating potential benefits in emotional connection and comfort growth. However, one study found no significant change in affective learning gains related to nursing students’ confidence and satisfaction (16). This may be due to the lack of nuanced empathy and personal connection in AI chatbots, which human instructors or peers can offer.

#### 3.5.2 Behavioral learning gains

Figure 3 illustrates the analysis of 18 identified affordances in nursing education, of which 9 pertain to behavioral learning gains, including assistance provision (*n* = 3), personalization (*n* = 4), distilling information (*n* = 1), and fostering familiarity (*n* = 1). Reported growth in behavioral learning gains encompasses self-directed learning (16), diagnostic skills (44), clinical practice (26), engagement (19), and chatbot usage frequency (28). The assistance provision (*n* = 3) by AI chatbots has been shown to enhance students’ self-directed learning and clinical practice (16, 26). For instance, Chen et al. (26) employed a qualitative approach using focus group interviews to explore how a chatbot-based history-taking training program benefited 22 third-year undergraduate nursing students, providing more opportunities for clinical practice in history-taking. Personalization (*n* = 4) by AI chatbots, through timely feedback, have shown a significant impact on students’ diagnostic skills, engagement, and frequency of chatbot use (19, 28, 44). For instance, Sáiz-Manzanares et al. (28) employed a monitoring tool to track the chatbot usage frequency among 57 students. The study found that master’s students demonstrated higher learning gains and used the chatbot more frequently than bachelor’s students.

#### 3.5.3 Cognitive learning gains

Figure 3 also illustrates the analysis of 18 identified affordances in nursing education, of which 14 pertain to cognitive learning gains, including assistance provision (*n* = 7), personalization (*n* = 3), human-like conversing (*n* = 2), and distilling information (*n* = 2). Improvements in cognitive learning gains include prior knowledge (28), learning achievement (18), academic performance (25), critical thinking (19), and problem-solving (19). The high-level digital affordances of AI chatbots, particularly in assistance provision (*n* = 7), effectively enhance nurses’ learning achievement, critical thinking, problem solving (19, 26). For instance, ANCOVA analysis in Chang et al. (19) revealed that 50 nursing students using a CIDI model-based ChatGPT learning method scored significantly higher in problem-solving skills than those using conventional learning methods. Similarly, personalization (*n* = 3) by AI chatbots was found to significantly enhance nursing students’ prior knowledge and academic performance (25, 28). For example, Sáiz-Manzanares et al. (28), employing a mixed-methods approach, found that students with moderate prior knowledge and those pursuing master’s degrees showed superior learning gains. However, research on using AI chatbots for distilling information (*n* = 1) and enhancing familiarity (*n* = 1) to support cognitive learning gains is still limited. Overall, seven studies used quantitative pre-post test methods to measure cognitive learning gains, while one study employed qualitative methods, specifically focus group interviews. The remaining studies adopted a mixed-methods approach. However, one study reported no statistically significant improvement in cognitive learning gains related to nursing students’ knowledge and clinical reasoning competency (16). This may be attributed to the inherent limitations of the AI chatbots in simulating complex clinical scenarios.

## 4 Discussion

We analyzed 25 studies on the application of chatbots in nursing education. The results indicate that researchers employed various chatbot technologies, including natural language processing (NLP), large language models, knowledge-based systems, and pattern matching. However, some studies did not specify the technical details of the technologies used. This finding supports existing research that calls for further exploration and optimization of chatbot technology in education (45, 46). Regarding the study duration, the lengths varied widely, ranging from a few hours to three months. This limits in duration reflects different considerations in evaluating the long-term effects of chatbots. This also highlights the need for more longitudinal studies to examine sustained effects (16, 47). In terms of research design, 12 quantitative studies focused on evaluating the impact of chatbots on nursing skills and knowledge. Three qualitative studies explored students’ perceptions, attitudes, and experiences with chatbots. Four mixed-methods studies evaluated multi-dimensional learning gains. However, another 6 studies did not specify their research methodology, which to some extent affected the transparency and generalizability of the reported results. This finding aligns with the existing view that chatbots have the potential to promote learners’ development and evaluation (31, 32, 47, 48).

### 4.1 RQ1: What digital affordances do AI chatbots provide in nursing education?

An analysis of 25 related studies, we identified five main categories of digital affordances of AI chatbots in nursing education, including assistance provision, personalization, human-like conversing, distilling information, and fostering familiarity. notably, assistance provision (*n* = 15) emerged as the most common type of digital affordances in nursing education (see Figure 3). These affordances not only enhance educational efficiency and learning motivation by increasing the interactivity and engagement of the learning experience but also support the development of autonomous learning, distance education, and personalized learning pathways (41). In contrast, AI chatbots providing high-level digital affordances such as personalization (*n* = 10), human-like conversing (*n* = 4), distilling information (*n* = 3), and fostering familiarity (*n* = 3) appear less frequently in nursing education (see Figure 3). This phenomenon might stem from the strong tradition of human-to-human interaction in nursing education, leading to resistance towards replacing or supplementing these interactions with AI chatbots (49). Specifically, affordance theory explains the potential functions of AI chatbots, while AI literacy determines how effectively nursing students engage with these affordances. However, previous studies on the main categories of digital affordances have revealed a lack of empirical support for the application of four high-level affordances in nursing education: facilitation, enriching information, context identification, and ensuring privacy. Potential reasons could include the following: Firstly, nursing education aims to cultivate professional competencies such as nursing skills, clinical experiences, and critical thinking, which current AI chatbots find challenging to simulate in authentic clinical nursing environments (50). Secondly, the veracity of professional information dispensed by AI chatbots in nursing education is yet to be ascertained (51). Furthermore, ethical concerns may arise from employing AI chatbots in delicate domains pertaining to patient care, privacy, and confidentiality (52–54). Lastly, certain individuals persist in favoring conventional teaching methodologies, posing conceptual obstacles to the adoption of novel technologies, as they are inclined to view chatbots solely as utilitarian devices rather than as collaborative educational partners (55).

### 4.2 RQ2: What types of evidence have been employed to assess the impact of AI chatbots on learning gains in nursing education?

We analyzed 18 related affordances identified in nursing education, most of which are associated with cognitive learning gains (*n* = 14), including assistance provision (*n* = 7), personalization (*n* = 3), human-like conversation (*n* = 2), and distilling information (*n* = 2). However, there is a lack of evidence supporting the role of related affordances provided by AI chatbots, such as error reduction, feedback, flow maintenance, and comfort growth in enhancing cognitive learning gains. A possible reason is that current studies using pre-post test and qualitative focus group interviews to measure cognitive learning gains (16, 24), which primarily assess students’ performances in areas such as prior knowledge (28), learning achievement (18), academic performance (25), critical thinking (19), and problem-solving (19), with improvements commonly observed. However, fewer studies (16) have reported significant changes in students’ affective learning gains (e.g., confidence and satisfaction) or cognitive learning gains (e.g., knowledge and clinical reasoning competency). This finding highlights the need for further research to elucidate the factors influencing these learning gains and to design more rigorous methods for their assessment. Notably, fewer studies have focused on behavioral learning gains (*n* = 9), encompassing assistance provision (*n* = 3), personalization (*n* = 4), distilling information (*n* = 1), and fostering familiarity (*n* = 1). This may be attributed to the fact that professional behavioral learning in nursing requires students to engage in hands-on practice in authentic or simulated clinical environments, mastering various nursing skills through practical operation and repeated training, such as patient care, surgical nursing, and intravenous infusion, rather than relying solely on dialogue interactions with AI chatbots (1). Furthermore, the inability of current AI chatbots to accurately simulate the pressure and complexity of authentic clinical scenarios may hinder their facilitative effect on behavioral learning gains. In other words, there is a lack of empirical research examining the processes and progress in learning gains facilitated by AI chatbots, making it challenging for educators to understand students’ learning trajectories and provide appropriate support. This imbalance across affective, behavioral, and cognitive domains should be considered when interpreting the findings and highlights the need for more balanced research across learning gains. Similarly, there is limited research on the impact of AI chatbot digital affordances on learning gains under different learning strategies, posing challenges for educators in implementing effective pedagogical interventions.

## 5 Implications

Firstly, we observed that high-level digital affordances of AI chatbots such as human-like conversing, distilling information, and fostering familiarity have a low frequency of application in nursing education. Additionally, previous studies have found a lack of empirical evidence supporting the application of four high-level digital affordances (facilitation, enriching information, context identification, and ensuring privacy) in nursing education. This aligns with existing research conclusions that AI currently struggles to realistically simulate clinical scenarios and provide reliable professional information (56). Furthermore, the introduction of AI may raise patient privacy and ethical concerns (57), and traditional teaching philosophies could hinder the adoption of new technologies (58). Overall, the use of AI chatbots in nursing education faces multifaceted challenges, including technological limitations, ethical considerations, and social acceptance (59). Concerns about AI replacing human interactions necessitate enhancing trust (60). When adopting new technologies in nursing education and practice, factors such as ethics, privacy, and professional development must be carefully weighed (61). This also addresses a key limitation in prior reviews, which often focus on technological features without adequately considering learners’ competencies and their role in shaping learning gains. Although this review focuses on student populations, perspectives from educators also provide valuable insights. For example, Sarıkahya et al. (62) highlighted that while ChatGPT enhances teaching efficiency and flexibility, concerns remain regarding students’ overreliance and reduced critical engagement. This suggests that both student and educator perspectives are important for understanding AI integration in nursing education. Therefore, realizing the potential of AI in nursing education requires a holistic approach that balances technological innovation, ethical compliance, mitigation of cognitive biases, and the cultivation of professional competencies.

Secondly, the existing literature provides limited empirical evidence supporting the influence of higher-level digital affordances on behavioral learning gains in nursing education. Therefore, future efforts should focus on developing AI chatbots capable of realistically replicating clinical environments, providing comprehensive assessments and feedback, and offering personalized support tailored to individual learners’ characteristics. Future research could employ methods such as sequence clustering, process mining, self-report questionnaires, trace data, and lag sequential analysis to provide clearer insights into the concept of “learning gains” for both teachers and students (28, 63–65). Furthermore, future research should establish new assessment frameworks to objectively measure the impact of digital affordances on different types of learning gains, and explore the use of concise, easy-to-understand tools suitable for large-scale application to measure the core dimensions of learning gains associated with the digital affordances of AI chatbots in nursing education (22). These dimensions include potential changes in knowledge, skills, attitudes, and values that may occur within the context of nursing education. Curriculum designers should align AI use with learning objectives, especially those related to clinical reasoning and professional judgment. For instance, analyzing the interaction process between students and AI chatbots can assist nursing educators in identifying key intervention points within the learning process, supporting the creation of more effective learning experiences, helping students understand their learning patterns and habits, and thus promoting the enhancement of self-regulated learning skills (62, 66).

## Data Availability

The original contributions presented in the study are included in the article/Supplementary material, further inquiries can be directed to the corresponding author.
