# Supplementary material for: Digital affordances of AI chatbots in nursing education: a systematic review of learning gains and gaps in the evidence
Source: Front Med (Lausanne). 2026 Apr 24;13:1832598. doi: 10.3389/fmed.2026.1832598 (PMC13152810; doi:10.3389/fmed.2026.1832598)
Supplement: Supplementary file 2 [file Supplementary_file_2.docx]

Additional file 2:

High-level digital affordances identified in the literature(Mygland et al., 2021)

| **High-level affordances** | **Definition** | **Related affordances** | **Reference** |
| --- | --- | --- | --- |
| Human-like Conversing | Chatbots offer users action possibilities for engaging in conversations, marking a shift in software interaction. They produce human-like messages, enabling natural language communication. Advances in natural language processing and conversational modeling have made interactions smoother. Chatbots can infer user intent, synthesize responses, and retain context for follow-up questions. | - Capture, Storage and Renderings of Voice Recordings | Lunberry & Liebenau (2021) |
|  |  | - Mimicry of Human-like Conversation Methods(Conversation mimicry) |  |
|  |  | - Mimicry of Human-like Conversational Elements |  |
|  |  | - Presentation of Human-like Message Content(Human-like content) |  |
|  |  |  |  |
|  |  | - Fostering team cohesion | Stoeckli et al. (2020) |
|  |  | - Enforcing discipline and compliance |  |
|  |  |  |  |
|  |  | - Socializing | Waizenegger et al. (2020) |
|  |  | - Mitigating boredom |  |
|  |  | - Simulating a human-like interaction |  |
|  |  | - Depending on the degree of anthropomorphism of virtual anthropomorphic advisors, they afford users to establish positive emotions (such as empathy) to increase users’ satisfaction during and after value co-creation in a U-shaped manner |  |
|  |  | - Through their anthropomorphic design, virtual anthropomorphic advisors help users overcome information disclosure barriers in value co-creation |  |
|  |  |  |  |
|  |  | - Hands-free and eyes-free use | Moussawi (2018) |
|  |  |  |  |
|  |  | - Communication | Lippert et al. (2019) |
|  |  |  |  |
| Assistance Provision | Chatbots assist employees and external audiences in everyday transactions, such as setting reminders, invoking software functions, and accessing information. In nursing education, they facilitate administrative tasks and provide personalized support, enhancing user engagement and efficiency. | - Receiving status notifications and updates | Stoeckli et al. (2020) |
|  |  | - Receiving real-time information (Live updates) |  |
|  |  | - Receiving metrics and key performance indicators |  |
|  |  | - Setting and getting reminders |  |
|  |  | - Setting and getting nudges/triggers to action |  |
|  |  | - Having messages processed and replaced |  |
|  |  | - Increasing visibility and ambient awareness |  |
|  |  | - Relieving employees from application switching |  |
|  |  | - Relieving employees from repetitive work |  |
|  |  |  |  |
|  |  | - Receiving status notifications and updates | Stoeckli et al. (2018) |
|  |  | - Receiving real-time information (Live updates) |  |
|  |  | - Receiving metrics and key performance indicators |  |
|  |  | - Getting reminded |  |
|  |  | - Getting nudges |  |
|  |  | - Getting a nudge to action and resolve it |  |
|  |  | - Invoking functionality |  |
|  |  | - Invoking functionality and making invocation visible |  |
|  |  |  |  |
|  |  | - Instantaneous solving of fact-based questions(Quick answers) | Waizenegger et al. (2020) |
|  |  | - Executing tasks |  |
|  |  | - Help-seeking for personal issues(Support seeking) |  |
|  |  | - Relief from mundane tasks |  |
|  |  | - Self-servicing |  |
|  |  |  |  |
|  |  | - Different affordances according to their unique combinations of material properties that influence value co-creation in smart services. | Knote et al. (2020) |
|  |  | - Afford users to spend more cognitive load on the actual value-creating task rather than on interacting with the system. |  |
|  |  | - Afford users to identify the technical object as an expert in a certain domain. |  |
|  |  |  |  |
|  |  | - Speedy assistance | Moussawi (2018) |
|  |  | - Usefulness |  |
|  |  |  |  |
|  |  | - Access relevant information(Data access) | Meske & Amojo (2020) |
|  |  | - Engage with application |  |
|  |  |  |  |
|  |  | - Minimising human error and maximising expertise(Error reduction) | Barnett et al. (2021) |
|  |  |  |  |
| Facilitation | Chatbots facilitate interactions between users and organizations by allowing users to query information or invoke functionalities without direct engagement with third parties. They unify access across systems, reducing task effort. In nursing education, they streamline administrative processes and enhance learning support. | - Capturing data in third party systems | Stoeckli et al. (2020) |
|  |  | - Querying information from third-party systems |  |
|  |  | - Invoking functions from third-party systems and make this invocation visible |  |
|  |  | - Unifying access to third-party systems |  |
|  |  | - Building rapid prototypes (F) |  |
|  |  |  |  |
|  |  | - General activity assistants afford smart service stakeholders to cocreate value through external integration, and, thus, shape affordances accordingly in a reciprocal and dynamic manner. | Knote et al. (2020) |
|  |  |  |  |
|  |  | - Contact relevant institutions | Meske & Amojo (2020) |
|  |  |  |  |
| Distilling Information | Chatbots help distill information by aggregating data and aiding users in understanding large volumes of information. They can also assist users in reflecting on their mood or mental state. In nursing education, chatbots streamline information processing and support mental health monitoring. | - Receiving aggregated information(Aggregated data) | Stoeckli et al. (2018) |
|  |  |  |  |
|  |  | - Ensuring information flow through uncoupling(Flow maintenance) | Stoeckli et al. (2020) |
|  |  | - Receiving aggregated information(Aggregated data) |  |
|  |  |  |  |
|  |  | - Afford users to effectively access and better understand large amounts of potentially consecutive information necessary for information-intensive value co-creation in a particular domain of interest. | Knote et al. (2020) |
|  |  |  |  |
|  |  | - Reflect own mood/mental state | Meske & Amojo (2020) |
|  |  |  |  |
| Enriching Information | Chatbots enhance information delivery by providing visual or additional text elements, thereby accelerating communication and enabling more effective connections. In nursing education, AI-driven information enrichment empowers chatbots to serve as more effective assistants in daily tasks. | - Having messages processed and enriched with additional information | Stoeckli et al. (2020) |
|  |  | - Having messages processed and visually enriched with user interface elements |  |
|  |  |  |  |
|  |  | - Voice facilitators afford the facility to complement or replace interaction modes other than voice in value co-creation with respect to specific user needs. | Knote et al. (2020) |
|  |  | - Voice facilitators afford the facility to complement other smart services through external integration that enable/shape new value co-creation possibilities. |  |
|  |  | - General activity assistants rely on continuous adaptation in affordance actualization processes through crowd data integration to improve value co-creation. |  |
|  |  |  |  |
| Context Identification | Chatbots can contextualize user queries and discussions, identifying specific information needs, providing responsive feedback, and guiding ongoing conversations. | - Consolidating information flow | Stoeckli et al. (2020) |
|  |  | - Facilitating feedback as reaction and discussions |  |
|  |  | - Separating organizational units |  |
|  |  |  |  |
|  |  | - Capturing data | Stoeckli et al. (2018) |
|  |  | - Querying information |  |
|  |  | - Having messages processed and replaced |  |
|  |  |  |  |
|  |  | - Afford users to explore a wide range of value co-creation possibilities for different purposes within their ecosystem. | Knote et al. (2020) |
|  |  | - Identify problem specific information | Meske & Amojo (2020) |
|  |  | - Access to other affordances |  |
|  |  | - Identify relevant institutions |  |
|  |  | - Identify others with similar problems |  |
|  |  | - Identify problem specific information |  |
|  |  |  |  |
| Personalization | Chatbots provide personalized experiences by adapting interactions to users, offering tailored responses, and adjusting tone and style. This personalization enhances user engagement and continuously improves as chatbots learn from interactions. | - Personal assistance | Waizenegger et al. (2020) |
|  |  |  |  |
|  |  | - SPAs provide different affordances for specified users or user groups, which in turn influences value co-creation in smart services. | Knote et al. (2020) |
|  |  |  |  |
|  |  | - Personalization and learning from interactions(Personalized learning) | Moussawi (2018) |
|  |  |  |  |
|  |  | - Interactivity | Lippert et al. (2019) |
|  |  | - Adaptivity |  |
|  |  | - Feedback |  |
|  |  | - Choice |  |
|  |  | - Nonlinear access |  |
|  |  | - Linked representations |  |
|  |  | - Open-ended learner input |  |
|  |  |  |  |
| Fostering Familiarity | Chatbots in nursing education leverage widespread familiarity with chat applications, requiring little prior experience. Users feel comfortable expressing their needs through familiar interactions, though this can also lead to dissatisfaction if expectations are not met. | - Emerging Tensions: Satisfaction and Disappointment | Moussawi (2018) |
|  |  | - Emotional connection |  |
|  |  | - Familiarity and Potential Improvement(Comfort growth) |  |
|  |  |  |  |
| Ensuring Privacy | Chatbots in nursing education implement privacy-preserving approaches and may control access to various functions. They often require users to disclose key information, necessitating careful privacy management during interactions. | - Adding gatekeepers that validate access to function of third-party systems | Stoeckli et al. (2020) |
|  |  |  |  |
|  |  | - Adding gatekeeper Stoeckli et al. (2020) | Stoeckli et al. (2018) |
|  |  |  |  |
|  |  | - Leveraging anonymity | Waizenegger et al. (2020) |
|  |  |  |  |
|  |  | - If the user is aware that the data-driven active observer collects context and usage data, information disclosure barriers (such as privacy and trust concerns) will negatively influence value co-creation in smart services. | Knote et al. (2020) |

**Reference:**

Barnett, A., Savic, M., Pienaar, K., Carter, A., Warren, N., Sandral, E., Manning, V., & Lubman, D. I. (2021). Enacting ‘more-than-human’ care: Clients’ and counsellors’ views on the multiple affordances of chatbots in alcohol and other drug counselling. *International Journal of Drug Policy*, *94*, 102910. https://doi.org/10.1016/j.drugpo.2020.102910

Knote, R., Janson, A., Söllner, M., & Leimeister, J. M. (2020). *Value Co-Creation in Smart Services: A Functional Affordances Perspective on Smart Personal Assistants*.

Lippert, A., Gatewood, J., Cai, Z., & Graesser, A. C. (2019). Using an Adaptive Intelligent Tutoring System to Promote Learning Affordances for Adults with Low Literacy Skills. In R. A. Sottilare & J. Schwarz (Eds.), *Adaptive Instructional Systems* (Vol. 11597, pp. 327–339). Springer International Publishing. https://doi.org/10.1007/978-3-030-22341-0_26

Lunberry, D., & Liebenau, J. (2021). Human or Machine? A Study of Anthropomorphism Through an Affordance Lens. In C. Metallo, M. Ferrara, A. Lazazzara, & S. Za (Eds.), *Digital Transformation and Human Behavior* (Vol. 37, pp. 201–215). Springer International Publishing. https://doi.org/10.1007/978-3-030-47539-0_15

Meske, C., Amojo, I., & Thapa, D. (2020, March). Understanding the Affordances of Conversational Agents in Mental Mobile Health Services. *In Forty-First International Conference on Information Systems(ICIS2020)*. India.

Moussawi, S. (2018). User Experiences with Personal Intelligent Agents: A Sensory, Physical, Functional and Cognitive Affordances View. *Proceedings of the 2018 ACM SIGMIS Conference on Computers and People Research*, 86–92. https://doi.org/10.1145/3209626.3209709

Mygland, M. J., Schibbye, M., Pappas, I. O., & Vassilakopoulou, P. (2021). Affordances in Human-Chatbot Interaction: A Review of the Literature. In D. Dennehy, A. Griva, N. Pouloudi, Y. K. Dwivedi, I. Pappas, & M. Mäntymäki (Eds.), *Responsible AI and Analytics for an Ethical and Inclusive Digitized Society* (Vol. 12896, pp. 3–17). Springer International Publishing. https://doi.org/10.1007/978-3-030-85447-8_1

Stoeckli, E., Dremel, C., Uebernickel, F., & Brenner, W. (2020). How affordances of chatbots cross the chasm between social and traditional enterprise systems. *Electronic Markets*, *30*(2), 369–403. https://doi.org/10.1007/s12525-019-00359-6

Stoeckli, E., Uebernickel, F., & Brenner, W. (2018). *Exploring Affordances of Slack Integrations and Their Actualization Within Enterprises –Towards an Understanding of How Chatbots Create Value*.

Waizenegger, L., Seeber, I., Dawson, G., & Desouza, K. C. (2020). *Conversational Agents—Exploring Generative Mechanisms and Second-hand Effects of Actualized Technology Affordances*.
