# Supplementary material for: Digital affordances of AI chatbots in nursing education: a systematic review of learning gains and gaps in the evidence
Source: Front Med (Lausanne). 2026 Apr 24;13:1832598. doi: 10.3389/fmed.2026.1832598 (PMC13152810; doi:10.3389/fmed.2026.1832598)
Supplement: Supplementary file 3 [file Supplementary_file_3.docx]

Additional file 3:

Evidence of chatbots digital affordances in nursing education

| Evidence of chatbots digital affordances in nursing education | | Affective | Behavioral | Cognitive |
| --- | --- | --- | --- | --- |
| Affordances related to Assistance Provision in nursing education (n = 8) | |  |  |  |
| Usefulness | The experimental group participants self‑directed learning (t = 2.72, p = .006) were significantly higher than those in the control group. (Han et al., 2022). In this case, all participants managed to perform CPR and one in three did so with high quality (Otero-Agra et al., 2022). The study revealed that the knowledge-based chatbot system improved students' academic performance, critical thinking, and learning satisfaction compared to traditional teaching methods(Chang et al., 2022b). Participants expressed that one area where chatbots could be used in are where students have to practise history taking(Kaur et al., 2021). The mobile chatbot-based learning approach (Mean = 88.58; SD = 11.02) had better results than the conventional didactic instructional approach (Mean = 60.51; SD = 15.01)(Chang et al., 2022a). the CIDI model-based ChatGPT system effectively boosted learners’ critical thinking, problem solving and learning enjoyment compared to conventional teaching(Chang et al., 2024). | √ | √ | √ |
| Speedy assistance | The chatbot, comprising introduction, main course, and conclusion stages, uses quizzes and questions to assess understanding, enhancing learning with feedback; a significant difference in self-directed learning was observed between experimental and control groups (Han et al., 2022). The CIDI model-based ChatGPT system offers valuable assistance to users in making appropriate choices and conducting systematic studies focused on specific learning content (Chang et al., 2024). | √ |  | √ |
| Executing tasks | The chatbot used in our study asked questions based on the students’ learnings from video lectures and offered feedback based on their answers (Han et al., 2022). In this learning task, the experimental group adopted the chatbot to learn and complete the learning sheet (Chang et al., 2022a). When a student talks to the chatbot, the “Analyzer” interprets the sentences submitted by the student, and searches for the relevant information from the knowledge base (Chang et al., 2022b). |  |  | √ |
| Live updates | The chatbot proficiently offers brachytherapy information; upon request, it briefs users and inquires about their interest in learning more (Chow et al., 2023). One significant advantage of the CIDI model based ChatGPT system is likely its capability to offer instant feedback, facilitating engagement and enabling learners to study efficiently according to their needs (Chang et al., 2024). | √ | √ | √ |
| Data access | The chatbot proficiently offers brachytherapy information; upon request, it briefs users and inquires about their interest in learning more (Chow et al., 2023). In the main course, students learn about nursing management and nursing interventions of electronic fetal monitoring devices through chatbot learning activities (Han et al., 2022). | √ |  | √ |
| Quick answers | For the ChatGPT group, scores rose from 53.7 pre-test to 82.85 post-test; the Termbot group showed a rise from 53.2 to 80.25, compared to the control group's increase from 54.45 to 62.5(Hsu, 2023). ChatGPT systems can benefit learners by acting as a tutor (Chang et al., 2024). |  |  | √ |
| Error reduction | The chatbot, enabling repetitive practice, offers students ample chances to attempt and err, a method many find beneficial for focusing on key clinical skills(Chen et al., 2023). |  | √ |  |
| Support seeking | Significant differences were noted in the SPSUCS scale items, except items 4 ('asking subject-related questions through the chatbot') and items 5 ('using the chatbot for issue resolution'), related to perceived benefits(Consuelo Saiz-Manzanares et al., 2023). | √ |  | √ |
| Affordances related to Personalization in nursing education (n = 4) | |  |  |  |
| Personalized learning | The knowledge-based chatbot system facilitated interactive and personalized learning, adapting to students' progress and needs (Chang et al., 2022b). Analyzing weekly time spent on medical terminology review evaluates how each learning tool impacted students' motivation (Hsu, 2023). These simulations can also be tailored to mimic a wide range of clinical situations, from common ailments to rare diseases, providing a safe and controlled environment for students to work on their clinical reasoning and decision-making skills(Riedel et al., 2023).The CIDI model-based ChatGPT system offered an interactive learning mode, enabling learners to acquire relevant knowledge based on their learning progress, thus providing personalized learning opportunities (Chang et al., 2024). | √ | √ | √ |
| Interactivity | The chatbot used in our study asked questions based on the students’ learnings from video lectures and offered feedback based on their answers (Han et al., 2022). When a student talks to the chatbot, the “Analyzer” interprets the sentences submitted by the student, and searches for the relevant information from the knowledge base (Chang et al., 2022b). The knowledge-based chatbot system facilitated interactive and personalized learning, adapting to students' progress and needs (Chang et al., 2022b). Analyzing weekly time spent on medical terminology review evaluates how each learning tool impacted students' motivation (Hsu, 2023). | √ | √ | √ |
| Feedback | Chatbot learning activities also provide feedback based on students’ responses and enhance their learning experience (Han et al., 2022). Analyzing weekly time spent on medical terminology review evaluates how each learning tool impacted students' motivation (Hsu, 2023). Through these simulations, students can practice their diagnostic skills, receive immediate feedback, and learn to navigate patient interactions effectively(Riedel et al., 2023). One significant advantage of the CIDI model based ChatGPT system is likely its capability to offer instant feedback, facilitating engagement and enabling learners to study efficiently according to their needs (Chang et al., 2024). | √ | √ |  |
| Adaptivity | Our study revealed that students with more prior knowledge achieved better learning outcomes, yet their chatbot usage frequency was unaffected. Notably, Master’s students outperformed Bachelor’s in learning outcomes and chatbot usage frequency(Saiz-Manzanares et al., 2023). |  | √ | √ |
| Affordances related to Human-like Conversing in nursing education (n = 2) | |  |  |  |
| Human-like content | The RT Bot reliably provides therapy information, responds to post-section inquiries, and courteously concludes interactions. It initiates conversations with greetings and name inquiries，and 70% of respondents view its content as above average in helpfulness and clarity (Chow et al., 2023). | √ |  | √ |
| Conversation mimicry | Nursing students noted the chatbot as a novel approach for enhancing their history-taking skills, distinct from traditional methods like role-playing and clinical practice (Chen et al., 2023). | √ |  | √ |
| Affordances related to Distilling Information in nursing education (n = 2) | |  |  |  |
| Flow maintenance | In the main course, students learn about nursing management and nursing interventions of electronic fetal monitoring devices through chatbot learning activities(Han et al., 2022). When a student talks to the chatbot, the “Analyzer” interprets the sentences submitted by the student, and searches for the relevant information from the knowledge base (Chang et al., 2022b).It would be good to use as an index to find out more about a presentation which could direct you to internal lectures and textbooks(Kaur et al., 2021). |  | √ | √ |
| Aggregated data | It would be good to use as an index to find out more about a presentation which could direct you to internal lectures and textbooks (Kaur et al., 2021). Learners can adapt their learning approach, accessing teaching materials that suit their preferences and repeatedly reviewing unfamiliar content (Chang et al., 2024). |  |  | √ |
| Affordances related to Fostering Familiarity in nursing education (n = 2) | |  |  |  |
| Emotional  connection | The chatbot greets the user and inquires about their name when initiating a conversation, concurrently showcasing its capability to provide information on brachytherapy (Chow et al., 2023). | √ | √ |  |
| Comfort growth | For introverted students lacking strong theoretical knowledge, chatbot usage reduces stress associated with trial and error. New technologies in nursing education enhance comfort and familiarity in clinical settings (Chen et al., 2023). It does mean we can practice in a safe space without it being stressful [...] I don’t think that it matters as much, because you’re not taking someone’s time and it would be less stressful (Kaur et al., 2021). | √ |  |  |

**Reference:**

Chang, C., Hwang, G., & Gau, M. (2022a). Promoting students’ learning achievement and self‐efficacy: A mobile chatbot approach for nursing training. *British Journal of Educational Technology*, *53*(1), 171–188. <https://doi.org/10.1111/bjet.13158>

Chang, C. Y., Kuo, S. Y., & Hwang, G. H. (2022b). Chatbot-facilitated nursing education. *Educational Technology & Society*, 25(1), 15-27.

Chang, C. Y., Yang, C. L., Jen, H. J., Ogata, H., & Hwang, G. H. (2024). Facilitating nursing and health education by incorporating ChatGPT into learning designs. *Educational Technology & Society*, 27(1), 215-230.

Han, J.W., Park, J., & Lee, H. (2022). Analysis of the effect of an artificial intelligence chatbot educational program on non-face-to-face classes: A quasi-experimental study. *BMC Medical Education*, *22*(1), 830. https://doi.org/10.1186/s12909-022-03898-3

Kaur, A., Singh, S., Chandan, J. S., Robbins, T., & Patel, V. (2021). Qualitative exploration of digital chatbot use in medical education: A pilot study. *Digital Health*, *7*, 20552076211038151. https://doi.org/10.1177/20552076211038151

Otero-Agra, M., Jorge-Soto, C., Cosido-Cobos, Ó. J., Blanco-Prieto, J., Alfaya-Fernández, C., García-Ordóñez, E., & Barcala-Furelos, R. (2022). Can a voice assistant help bystanders save lives? A feasibility pilot study chatbot in beta version to assist OHCA bystanders. *The American Journal of Emergency Medicine*, 61, 169-174.

Chen, Y., Lin, Q., Chen, X., Liu, T., Ke, Q., Yang, Q., Guan, B., & Ming, W.-K. (2023). Need assessment for history-taking instruction program using chatbot for nursing students: A qualitative study using focus group interviews. *Digital Health*, *9*, 20552076231185435. https://doi.org/10.1177/20552076231185435

Chow, J. C., Wong, V., Sanders, L., & Li, K. (2023, August). Developing an AI-assisted educational chatbot for radiotherapy using the IBM Watson assistant platform. In Healthcare (Vol. 11, No. 17, p. 2417). MDPI.

Sáiz-Manzanares, M. C., Marticorena-Sánchez, R., Martín-Antón, L. J., Díez, I. G., & Almeida, L. (2023). Perceived satisfaction of university students with the use of chatbots as a tool for self-regulated learning. *Heliyon*, *9*(1).

Hsu, M.H. (2023). Mastering medical terminology with ChatGPT and Termbot. *HEALTH EDUCATION JOURNAL*. https://doi.org/10.1177/00178969231197371

Riedel, M., Kaefinger, K., Stuehrenberg, A., Ritter, V., Amann, N., Graf, A., ... & Meyer, B. (2023). ChatGPT’s performance in German OB/GYN exams–paving the way for AI-enhanced medical education and clinical practice. *Frontiers in Medicine*, 10, 1296615.
